# Deep immune profiling of intrahepatic cholangiocarcinoma with CODEX multiplexed imaging

**DOI:** 10.1097/HC9.0000000000000632

**Published:** 2025-02-19

**Authors:** Marina Baretti, Soumya Shekhar, Vaibhav Sahai, Daniel Shu, Kathryn Howe, Valerie Gunchick, Naziheh Assarzadegan, Emma Kartalia, Qingfeng Zhu, Elsa Hallab, Archit Sheth-Shah, Aya Kondo, Nilofer S. Azad, Mark Yarchoan

**Affiliations:** 1 Department of Medical Oncology, Sidney Kimmel Comprehensive Cancer Center, Johns Hopkins University, Baltimore, Maryland, USA; 2 Division of Hematology-Oncology, Department of Internal Medicine, University of Michigan, Rogel Cancer Center, Ann Arbor, Michigan, USA; 3 Department of Pathology, Johns Hopkins University School of Medicine, Baltimore, Maryland, USA; 4 Enable Medicine, Menlo Park, California, USA

**Keywords:** biliary tract cancer, cholangiocarcinoma, CODEX, FGFR2, IDH1

## Abstract

**Background:**

Intrahepatic cholangiocarcinoma (iCCA) may be genomically subclassified by the presence of potentially actionable molecular aberrations, of which pathogenic alterations in isocitrate dehydrogenase (IDH)1 and fibroblast growth factor receptor (FGFR)2 are the most frequently observed. The impact of these molecular alterations on the tumor immune microenvironment remains incompletely understood.

**Methods:**

We performed a high-parameter spatial immune phenotyping of iCCA samples with pathogenic FGFR2 or IDH1 alterations and FGFR2/IDH1 wild-type controls at the single-cell level using CO-Detection by indEXing.

**Results:﻿:**

A total of 24 tumors were examined. Tumors with FGFR2 alterations were characterized by fewer CD8+ T cells and “M2-like” macrophages but higher levels of polymorphonuclear myeloid-derived suppressor cells as compared to FGFR2 wild-type tumors. Spatial relationships between polymorphonuclear myeloid-derived suppressor cells and multiple other cell types in the tumor microenvironment (including tumor cells, CD4+, and CD8+ T cells) were enriched in tumors with FGFR2 alterations. Tumors with IDH1 mutations had a trend toward more fibroblasts and were characterized by a closer proximity of tumor cells to CD4+ T cells, and between macrophages and multiple structural tumor microenvironment components as compared to other subtypes.

**Conclusions:**

iCCAs with pathogenic FGFR2 fusions/rearrangements and IDH1 mutations have distinct immunophenotypes. Tailoring immunotherapeutic approaches to specific molecular subsets could improve treatment outcomes across the divergent molecularly defined iCCA subtypes.

## INTRODUCTION

Cholangiocarcinomas (CCA) are heterogeneous biliary epithelial tumors classified into intrahepatic (iCCA), perihilar, and distal subtypes based on their anatomic location.^1^ The median overall survival (OS) for patients with advanced CCA is approximately 12 months.^2^,^3^ Immune checkpoint inhibitor (ICI) therapy has recently become a focus of CCA treatment based on phase 3 clinical trials showing modestly improved survival with chemotherapy plus ICIs versus chemotherapy alone.^4^ However, CCA is generally characterized as an immune-resistant tumor, and response rates to ICI therapy alone are low in unselected CCA (in a range of 5%–10% in the largest prospective cohort studies).^5^,^6^,^7^,^8^ CCAs are broadly characterized by a non–T-cell-infiltrated tumor microenvironment (TME) with a preponderance of immunosuppressive innate immune cells such as tumor-associated macrophages (TAM) and myeloid-derived suppressor cells (MDSCs).^9^ An enhanced comprehension of the immunobiology of CCA is essential to identify biomarkers that identify patients likely to respond to ICI and to develop effective combination immunotherapeutic strategies.

CCA has a well-described molecular heterogeneity. Hotspot gain-of-function mutations in isocitrate dehydrogenase (IDH1) occur almost exclusively in iCCA (approximately 15% of iCCA).^10^

These hotspot gain-of-function mutations cause the IDH enzyme to aberrantly convert α-ketoglutarate to D-2-hydroxyglutarate using NADPH as a cofactor, which competitively inhibits enzymes that regulate epigenetics, DNA repair, metabolism, and other processes. IDH mutations also impair the activity of the TET family of DNA dioxygenases (which are α-ketoglutarate dependent), resulting in a decrease of cytosine hydroxymethylation with a concurrent increase of CpG island and histone methylation.^11^,^12^,^13^ This process suggests global epigenetic deregulation within the transcriptional machinery. Ivosidenib (AG120) is an oral and selective small molecule mIDH1 inhibitor that can significantly decrease intracellular and circulating R-2HG and was recently approved for advanced or metastatic mIDH1 iCCA in a second-line setting.^14^ However, the final results of the phase III ICC trial indicated a statistically significant but rather limited clinical benefit: AG120 produced only a 2% objective response rate and a progression-free survival benefit of <2 months (median 2.7 vs. 1.4 mo).^14^ Therefore, mIDH1 CCA remains a key area of unmet clinical need and a potential pathway for novel agent approval.

The FGF/FGFR (fibroblast growth factor receptor) signaling is implicated in cell proliferation, differentiation, angiogenesis, and intracellular survival through the RAS-dependent mitogen-activated protein kinase, phosphatidylinositol 3-kinase/Akt/mechanistic target of rapamycin pathways, Janus kinase signal transducer and activator of transcription pathway, and phospholipase Cγ pathway.^15^,^16^,^17^ In iCCAs, the predominance of FGFR aberrations has been highlighted in the gene encoding for FGFR2, and in particular, a majority of gene fusions or rearrangements have been identified, with amplifications and mutations reported as less frequent events. These FGFR fusions are almost exclusively seen in iCCA in approximately 10% of cases.^18^ FGFR2 fusion-positive iCCAs have been suggested to constitute a distinct and unique molecular subtype. Accordingly, in recent years, the role of FGFR-directed therapies has been explored in phase I to III clinical trials, and the Food and Drug Administration approved 3 main molecules for managing previously treated, unresectable, locally advanced, or metastatic CCA with an FGFR2 fusion or another rearrangement.^19^,^20^,^21^

Alterations in IDH1 and FGFR2 rarely co-occur, and cluster analyses of CCA suggest that these alterations are defining features of distinct iCCA subtypes, with unique physiology and prognosis.^22^,^23^,^24^ In preclinical models, the introduction of distinct genomic drivers of iCCA can have profound effects on the TME.^25^ Recent work from our group and others utilizing bulk transcriptomic data from hundreds of CCA tumors provides initial evidence that CCA tumors with IDH1 or FGFR2 pathogenic alterations have distinct TMEs.^22^,^24^,^26^ However, the contribution of alterations in these driver pathways (FGFR2 or IDH1) and their impact on remodeling the TME in CCA has not been fully explored and investigated at the protein level. Here, we use CO-Detection by indEXing (CODEX) to examine the relationships between pathologic alterations in FGFR2 and IDH1 and the spatial components of the iCCA TME.

## METHODS

### Patient selection and evaluation

We retrospectively collected clinically annotated tumor samples from 24 iCCA patients treated at the Johns Hopkins Hospital between 2011 and 2021 and at the University of Michigan between 2000 and 2020. These samples included 7 FGFR2 fusion/rearrangement positive iCCA (mFGFR2), 9 IDH1 mutant iCCA (mIDH1), and 8 FGFR2/IDH1 wild-type (WT) controls with molecular alterations identified using different Clinical Laboratory Improvement Amendments-certified laboratory platforms. For the staging of iCCA, we adopted a practical staging approach where the emphasis is on operability and considerations of risk of local-regional and distant disease recurrence.^27^ Histological details of the tumors were retrieved from the archived pathological reports. Clinical data were obtained from patient records. All research was conducted in accordance with both the Declarations of Helsinki and Istanbul. ﻿The study was approved by the Johns Hopkins Institutional Research Board (IRB002961130) and the University of Michigan Institutional Research Board (HUM00149617). A consent waiver from the IRB was obtained.

### CODEX

We optimized CODEX for use in formalin-fixed paraffin-embedded tissue to study archival human samples.^28^ One to 3 cores of 0.6 mm diameter from each biopsy sample were digitally annotated and compiled into a tissue microarray. For 12 patients, we had one core; for 10 patients, we had 2 cores; and for 2 patients, we had 3 cores. We used a 38-marker antibody panel to study our sample specimens: AXL, CD107a, CD117, CD11b, CD134, CD14, CD141, CD15, CD163, CD183, CD197, CD20, CD21, CD31, CD34, CD3e, CD4, CD45, CD45RA, CD45RO, CD56, CD68, CD8, GATA3, Granzyme B, HLA-DR, ICOS, IDH1, PD1, PDL1, protein gene product 9.5, pan-cytokeratin (PanCK), Podoplanin, SPP1, Siglec8, Tbet, Vimentin, and anti-smooth muscle antibody (Supplemental Table S1, http://links.lww.com/HC9/B887). All samples were prepared, stained, and acquired following CODEX User Manual Rev C (https://www.akoyabio.com), as described.^29^

### Cell phenotyping

The Uniform Manifold Approximation and Projection (UMAP) was then run on the dataset using uwot which is the R implementation of UMAP.^30^ Our IDH1/FGFR2-altered samples were evaluated for the 38 biomarkers listed previously. The biomarkers were selected to first run UMAP on the scaled dataset and then run clustering using the Leiden algorithm. The resulting UMAP was then plotted and colored by clusters identified by unsupervised clustering (Supplemental Figure S1, http://links.lww.com/HC9/B887). There was an even distribution of cells from all regions in each cluster, indicating minimal batch effect. A heatmap was created to identify cluster-specific biomarker expression profiles, which allowed for the unique identification of specific cell types. The final clusters were then integrated as phenotypes into an annotation set uploaded to the Enable Medicine platform.

### Quality control

Biomarker staining quality was evaluated by an immunologist for specificity and signal-to-noise characteristics as is standard. Each biomarker was scored for staining performance using the following criteria: 3, highest quality with cell-type and subcellular specificity, low background signal; 2, good quality with cell-type and subcellular specificity, possibly some background signal; 1, some cell-type and subcellular specificity may be detected, background signal may be high; 0, no biomarker specificity observed, high background signal; ND, not determined, stain specificity could not be determined. There were several positive controls used to validate the CODEX method itself, which included normal human tonsils, ovaries, pancreas, spleen, kidney, and liver.

### Cellular quantification

Unpaired two-sample *t* tests using Welch correction were run to compare the proportion of specific cell types between our samples—FGFR2 compared to others or IDH1 compared to others. Secondary 3 group analysis (FGFR2, IDH1, and WT iCCA) was done using one-way ANOVA with a post hoc comparison done using Tukey multiple comparison tests. These analyses were done using Prism version 10.0.2 for Mac (GraphPad Prism, San Diego, CA). A *p*-value<0.05 was considered to be statistically significant.

### Cellular interactions

To identify the strength and relationship of cellular relationships, we first calculated the cell interaction through the Enable Cloud Platform. In the platform, cell interaction was defined as contact between the Voronoi expansions from each cell’s centroid. This was not normalized for the frequency of each cell type, so it could be driven by both interactions and cell frequency. We aggregated the data by sample so that all regions belonging to the same sample were considered a single entity. This aggregation was performed because we had access to multiple samples from the same patient. The output was an interaction proportion, which was defined as the total number of cells from one subtype that interacted with cells from another subtype. These interaction proportions were then plotted on an interaction chord diagram where the strength of the relationship was denoted by the width of the arc. The chord diagrams were created using R version v4.0.2.

### Cellular spatial neighbor distance and network plots

The Enable Cloud Platform’s Spatial Neighbor Distance tool was used to conduct a cellular neighborhood analysis. We created runs between 2 comparison groups. The extension was able to find a specific “from” cell, find the nearest neighbor of the “to” cell type, and compute the distance. This was done across the entire data set from the Enable platform. Each cell of the “from” type was annotated with the distance to its nearest neighbor of the “to” type. The annotations were downloaded, and weighted means were calculated for each sample. One-way ANOVA was done comparing three subgroups (FGFR2, IDH1, and WT iCCA) with a post hoc comparison done using Tukey multiple comparison tests. The analyses and violin plots were created using R version v4.0.2. A *p*-value of <0.05 was considered to be statistically significant.

For network plots, the CODEX data were visualized using the Enable Medicine web interface. Additional analyses were performed by downloading data from the Enable Medicine website and using R version 4.2.2 and the R packages circlize v0.4.15 and qgraph v1.9.3.

### Data and code availability

All CODEX-generated data and correlative analysis code will be made available upon publication. The code will be available on GitHub and is currently available upon request.

## RESULTS

### Categorization of patient samples by molecularly defined subsets

**FIGURE 1:**
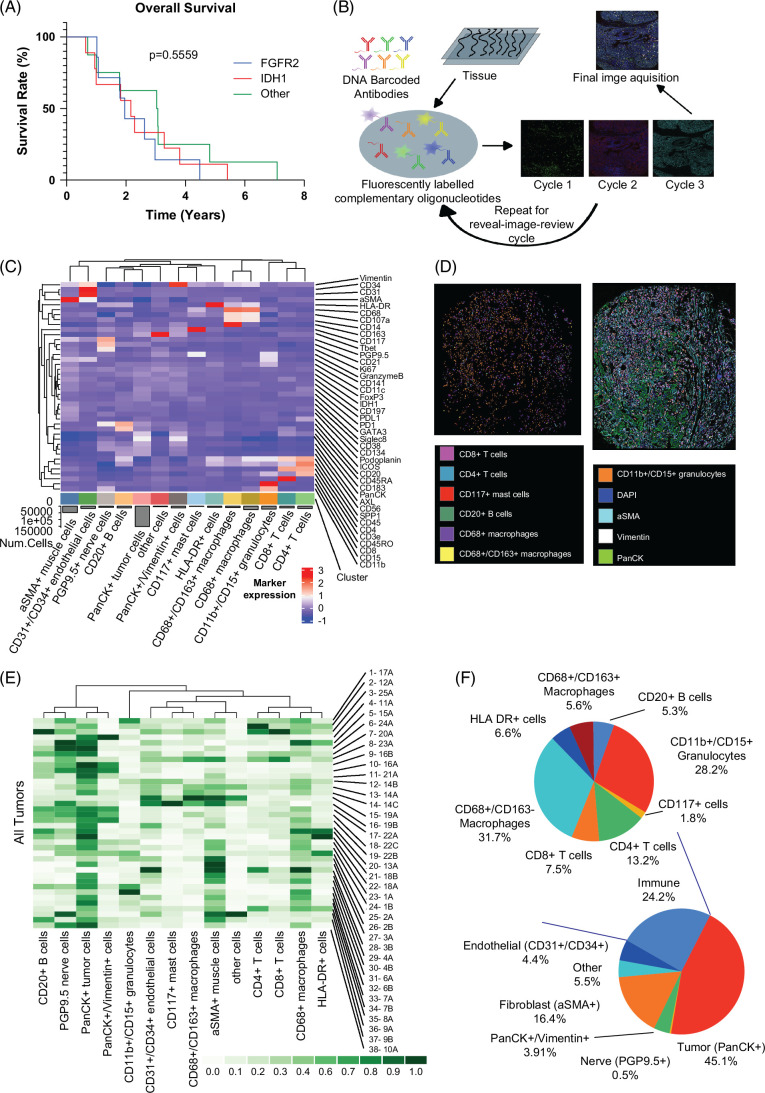
Characterization of the sample population by molecularly defined subsets. (A) Kaplan-Meier. Survivorship curve showing the overall survival (OS) for patients in each of the three subtypes. (B) CODEX workflow diagram. (C) Heatmap showing the biomarker expression of each cluster which was identified with UMAP. (D) CODEX images highlighting the cell distribution (top) with stroma overlay (bottom) with cell types identified by color, scale 250 μm. (E) Heatmap comparing relative frequencies of immune cell types across all samples, scale with the frequency of expression at the bottom. (F) Pie chart with the figure on the left highlighting the breakdown of cell subtypes found in the TME and image on the right showing the breakdown of immune cell subtypes. Abbreviations: aSMA, anti-smooth muscle antibody; BRCA, breast cancer gene; CODEX, co-detection by indexing; FGFR, fibroblast growth factor receptor; HLA, human leukocyte antigen; IDH, isocitrate dehydrogenase; PanCK, pan-cytokeratin; PGP, protein gene product.

Table 1 shows patient demographics and molecular characteristics of the entire cohort subdivided by molecular subtypes, with a focus on FGFR2 activating fusions or rearrangements (n=7, 29.2%) and IDH1 gain-of-function mutations (n=9, 37.5%). FGFR2 fusions or rearrangements were mutually exclusive with IDH1 pathogenic mutations in the study cohort. The average age was 55 years (range: 25–77 y). Most tumor samples (n=22, 91.8%) were obtained prior to systemic treatment; however, in 2 cases, samples were collected after receiving systemic treatment with gemcitabine and cisplatin. At the time of diagnosis, almost one-third (n=7, 29.2%) of patients had metastatic disease, 9 (37.5%) were considered resectable, 1 (14.3%) borderline resectable, and 7 patients (29.2%) were locally advanced, with a similar distribution across the molecular subtypes. The median OS for our patient population was 26.7 months (range: 7.2–85 mo), and OS was similar across the 3 subgroups (Table 1, Figure 1A).

**TABLE 1:** Demographic table defining our patient population

Variable	FGFR2 (N=7)	IDH1 (N=9)	WT (N=8)
Age at diagnosis (y)	Frequency (%)	Frequency (%)	Frequency (%)
20–40	1 (14.3)	1 (11.1)	1 (12.5)
41–60	5 (71.4)	4 (44.4)	4 (50.0)
61–80	1 (14.3)	4 (44.4)	3 (37.5)
Sex			
M	5 (71.4)	4 (44.4)	4 (50.0)
F	2 (28.6)	5 (55.6)	4 (50.0)
Stage at diagnosis			
Resectable	3 (42.9)	4 (44.4)	2 (25.0)
Borderline Resectable	1 (14.3)	0	0
Locally Advanced	1 (14.3)	2 (22.2)	4 (50.0)
Metastatic	2 (28.6)	3 (33.3)	2 (25.0)
Overall survival (y)			
0–2	3 (42.9)	4 (44.4)	3 (37.5)
2–4	3 (42.9)	4 (44.4)	3 (37.5)
4+	1 (14.3)	1 (11.1)	2 (25.0)
Surgical resection			
Yes	6 (85.7)	6 (66.7)	6 (75.0)
No	1 (14.3)	3 (33.3)	2 (25.0)
Treatment before sample collection			
Yes	1 (14.3)	0	1 (12.5)
No	6 (85.7)	9 (100)	7 (87.5)

*Note:* WT tumors include breast cancer gene (*BRCA2*) (n=1), *IDH2* (n=1). *PBMR1* (n=2), *KRASG12D* (n=1), *TP53* (n=1), *RB1* (n=1) mutations, and *MET* copy number (n=1).

Abbreviations: FGFR, fibroblast growth factor receptor; IDH, isocitrate dehydrogenase; WT, wild type.

### Identification and quantification of major immune cell subtypes in the iCCA TME

We optimized and applied a 38-marker antibody panel to characterize the composition, spatial organization, and immune landscape of the TME in formalin-fixed paraffin-embedded iCCA by CODEX (Figure 1B). Using UMAP clustering, we stratified cells into 14 cell types with 7 immune cell subtypes (Figure 1C and Supplemental Figure S1, http://links.lww.com/HC9/B887). The immune cell types included CD20+ B cells, CD4+ T cells, CD8+ T cells, CD11b+/CD15+ granulocytes, CD68+ macrophages, and CD117+ mast cells. To measure monocyte/macrophage phenotype, we examined activated M1/M2 polarity using CD163 expression. While HLA-DR+ cells could represent dendritic cells, we noted a variable range of CD11c expression, rendering further characterization of these cells difficult based on membrane protein expression. Similarly, clustering was unable to distinguish PanCK+/Vimentin+ cells from tumor cells, fibroblasts, or stromal cells. A representative eleven-color overlay image showing structural, lymphoid, myeloid, and tumor cell markers in iCCA tissue microarray core is shown in Figure 1D and Supplemental Figure S2, http://links.lww.com/HC9/B887. Markers for T-cell subsets (CD4, CD8), macrophages (CD68, CD163), granulocyte cells (CD11b), tumor cells (PanCK), vasculature (CD31), and epithelium (cytokeratin) were clearly visualized in the CODEX fluorescent images.

Across all 24 samples profiled, the most abundant cell type was tumor cells (45.1%). Fibroblast cells (αSMA+) were also prevalent, making up 16.4% of the overall TME. Immune cells comprised less than one-fourth (24.2%) of all cells within the TME. Most immune components of the TME were innate immune cells. Macrophages were the most prevalent immune cell type observed, representing 37.3% of immune cells, followed by CD11b+/CD15+ granulocytes (28.2%). CD8+ T cells comprised 7.5% of cells within the immune cluster (1.8% of cells in the TME overall) (Figure 1E, F).

### FGFR2 fusions or rearrangements are associated with reduced CD8+ T-cell infiltration and abundance of CD11b+/CD15+ granulocytes

**FIGURE 2:**
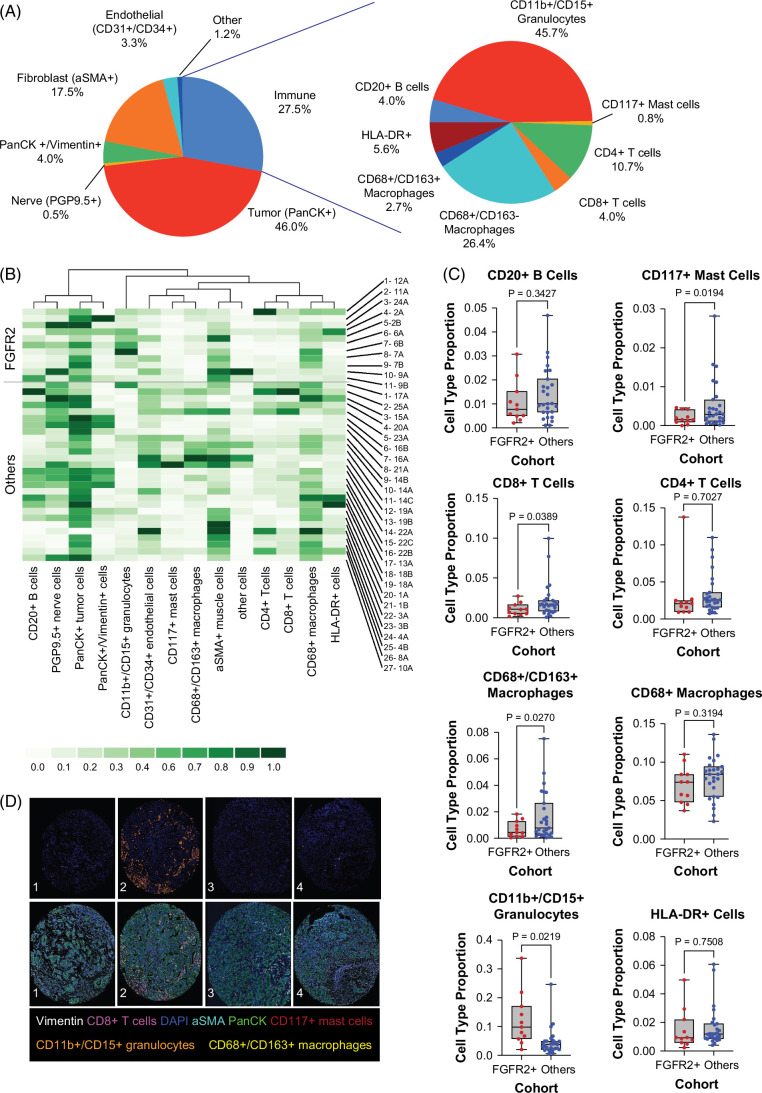
Comparisons of the TME of FGFR2+ tumors with other molecular subtypes. (A) Pie chart highlighting the breakdown of cell types (left) and immune cell subtypes (right) in FGFR2+ tumors. (B) Heatmap comparing relative frequencies of immune cell types broken down by FGFR2+ tumors (top half) and other molecular subtypes (bottom half). (C) Box and whisker plots comparing the proportion of immune cells in FGFR2+ tumors compared to other subtypes. P-values from unpaired t-test using Welch’s correction. (D) Cell type (top) and CODEX images (bottom) of representative FGFR2+ samples highlighting immune cell subtypes. Panel 1: CD8+ T cells; Panel 2: CD11b+/CD15+ granulocytes; Panel 3: CD117+ mast cells; Panel 4: CD68+/CD163+ macrophages. Abbreviations: aSMA, anti-smooth muscle antibody; FGFR, fibroblast growth factor receptor; PGP, protein gene product; HLA, human leukocyte antigen.

We subsequently investigated the distinctive features of the tumor immune microenvironment of tumors with FGFR2 fusions or rearrangements (FGFR2+ cohort) as compared to tumors without FGFR2 fusions or rearrangements (FGFR2 WT cohort). The most abundant cell types within the tumor immune microenvironment in the FGFR2+ cohort were CD11b+/CD15+ granulocytes, followed by macrophages (Figure 2A, B). Based on protein-membrane expression (high CD15) and morphology, these CD11b+/CD15+ granulocytes were classified as polymorphonuclear myeloid-derived suppressor cells (PMN-MDSC). The proportion of tumor cells, endothelial cells, and nerve cells was similar to the overall cohort (Supplemental Figure S3, http://links.lww.com/HC9/B887). The abundance of CD11b+/CD15+ granulocytes was significantly more in the FGFR2+ cohort as compared to the FGFR2 WT cohort (*p*<0.05). Conversely, FGFR2+ tumors had a significantly lower abundance of CD8+ T cells, CD117+ mast cells, and CD68+/CD163+ macrophages (all *p*<0.05). There was no significant difference in the frequency of CD20+ B cells or CD4+ T cells (Figure 2B–D and Supplemental Figures S3–S7, http://links.lww.com/HC9/B887). These trends were confirmed on a secondary analysis comparing all 3 groups separately (Supplemental Figure S3, http://links.lww.com/HC9/B887). These data show a relationship between FGFR alterations, a non–T-cell inflammatory phenotype, and enrichment in suppressive myeloid cells. The CODEX platform allowed limited analysis of T-cell subsets, and we were able to look at subsets, including regulatory T cells, helper T cells, and double negative T cells to see if there were differences among the subgroups. While there were no significant differences, the analyses confirmed the presence of fewer double negative T cells in FGFR2+ tumors compared to other subtypes and also showed a trend toward more regulatory T cells in IDH1+ tumors (Supplemental Figure S8A, http://links.lww.com/HC9/B887).

We also investigated T-cell functional markers, including Granzyme B (GZMB), PD1, PDL1, ICOS, and Tbet. There was significantly more GZMB expression in CD4+ T cells in FGFR2+ iCCA compared to both mIDH1 (*p*<0.05) and other subtypes (*p*<0.05) (Supplemental Figure S9B, http://links.lww.com/HC9/B887). No other significant differences were found among molecular subtypes when looking at PD(L)1, ICOS, SPP1, or Tbet expression on T-cell populations (Supplemental Figure S9B, http://links.lww.com/HC9/B887).

### Immune populations in the mIDH1 iCCA TME

**FIGURE 3:**
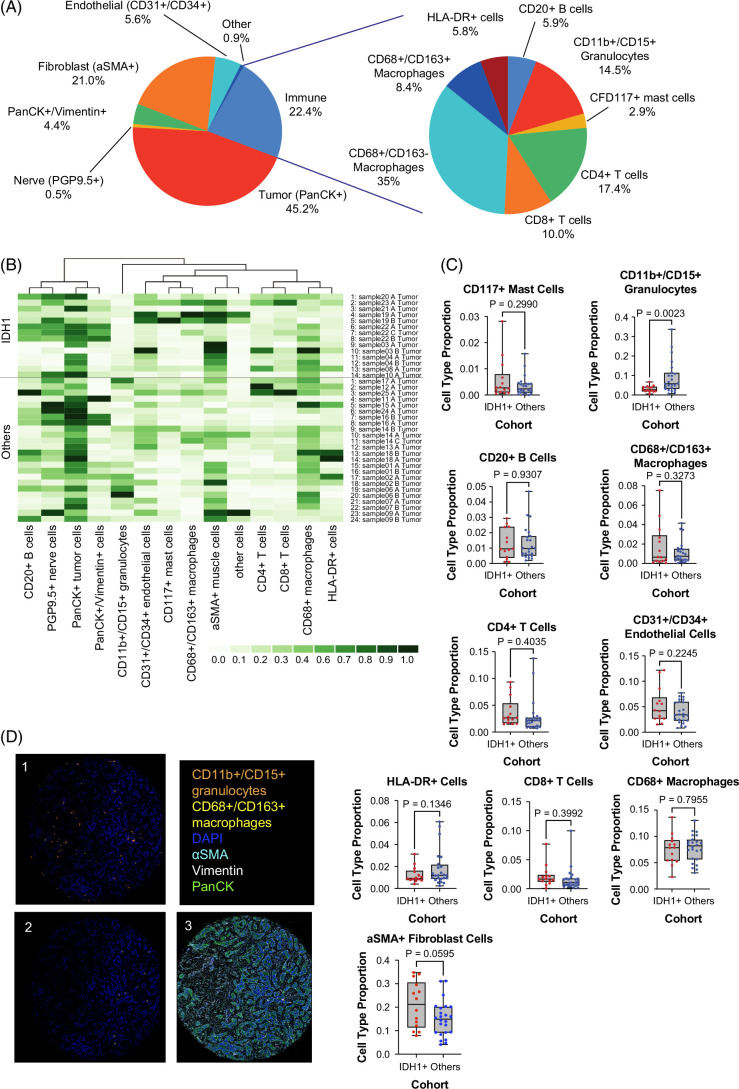
Comparisons of the TME of IDH1+ tumors with other molecular subtypes. (A) Pie chart highlighting the breakdown of cell types (left) and immune cell subtypes (right) in IDH1+ tumors. (B) Heatmap comparing relative frequencies of immune cell types with IDH1+ tumors (top half) and other molecular subtypes (bottom half). (C) Box and whisker plots comparing the proportion of cell subtypes in IDH1+ tumors compared to other subtypes. P-values from unpaired t-test using Welch’s correction. (D) CODEX and (panel 1 and 2) cell type images (panel 3) of representative IDH1+ samples highlighting immune cell subtypes. Panel 1: CD11b+/CD15+ granulocytes; Panel 2: CD68+/CD163+ macrophages. Abbreviations: aSMA, anti-smooth muscle antibody; HLA, human leukocyte antigen; IDH, isocitrate dehydrogenase; PanCK, pan-cytokeratin; PGP, protein gene product.

We next evaluated the TME of mIDH1 tumors (IDH1+ cohort) as compared to the tumors without pathogenic IDH1 mutations (IDH1 WT cohort) (Figure 3 and Supplemental Figure S3, http://links.lww.com/HC9/B887). Similar to the overall cohort, tumor cells were the most abundant cell type in the IDH1 cohort, followed by immune cells and fibroblasts (Figure 3A). Within the immune cluster, the IDH1+ cohort demonstrated a lower abundance of CD11b+/CD15+ granulocytes as compared to the IDH1 WT cohort (*p*<0.05) (Figure 3B, C). This difference was driven by the higher abundance of CD11b+/CD15+ granulocytes in the FGFR2+ cohort, as the abundance of CD11b+/CD15+ granulocytes in the IDH1+ cohort was similar to the FGFR2 WT/IDH1 WT cohort (data not shown). The IDH1+ cohort also had significantly more fibroblasts compared to the other subgroups, which was confirmed when analyzing the three groups separately (Figure 3C and Supplemental Figure S3, http://links.lww.com/HC9/B887).

### Spatial relationships among the cell types are distinct in molecularly defined subsets of iCCA

**FIGURE 4:**
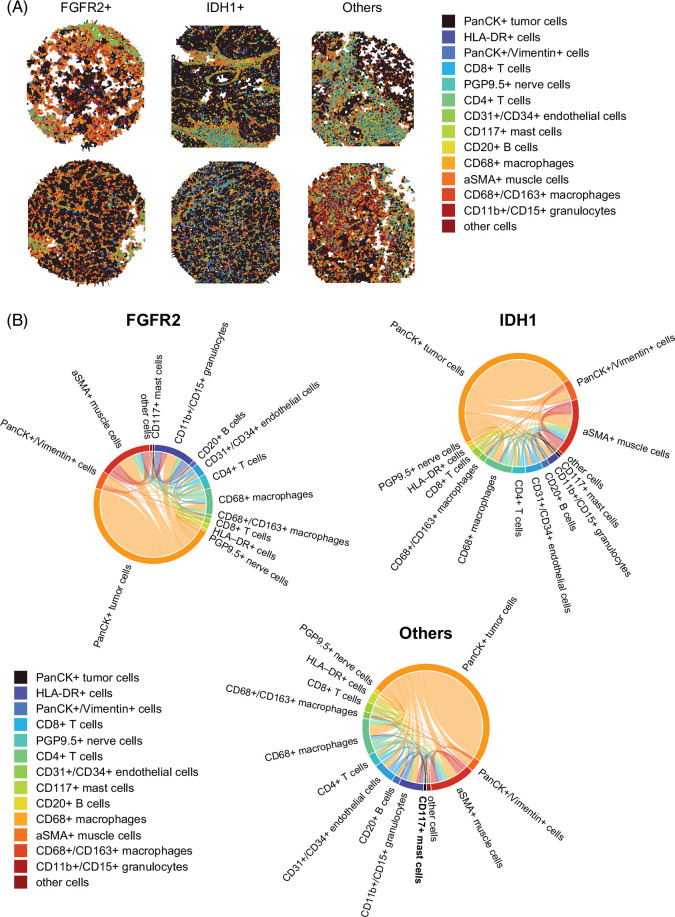
Analysis of the relationships between cell subtypes in tumor microenvironment of all subtypes. (A) Representative. Voronoi tessellations of 2 FGFR2+ samples (left), 2 IDH1+ samples (middle), and 2 other samples (right). (B) Chord diagrams were created from an analysis of CODEX images. The frequency of the cell sample is shown with the hump, and the strength of the relationship is highlighted with the arc. Abbreviations: aSMA, anti-smooth muscle antibody; FGFR, fibroblast growth factor receptor; HLA, human leukocyte antigen; IDH, isocitrate dehydrogenase; PanCK, pan-cytokeratin; PGP, protein gene product.

We then analyzed spatial coordinates between different cell subsets and visualized their location within the overall TME architecture in the molecularly defined subsets of iCCA. First, Voronoi tessellations were used to visualize a map of immune cell-type positions. These diagrams allowed for individual cells to be rendered into partitioned polygons based on the proximity of the neighboring cells to distinguish regions composed of homogeneous cell types from regions composed of heterogeneous cell types. The majority of our selected samples had heterogeneous regions of tumor cells intermixed with immune cells. However, a few immune-rich regions were demonstrable as distinct aggregates of lymphoid cells, especially T cells and macrophages in mIDH1 and T cells and granulocytes in mFGFR2 iCCA (Figure 4A).

We then created chord diagrams to further analyze the intercellular interactions that distinguish the different molecular subsets. This diagram allows for the visual representation of the interactions between cell clusters: the frequency of the cell sample is shown with the hump, and the strength of the relationship is highlighted with the arc. Integrative analysis showed that the most dominant pairwise cell-cell contacts in mFGFR2 iCCA were between tumor cells and CD11b+/CD15+ granulocytes as well as between tumor cells and αSMA+ fibroblasts and CD68+/CD163- macrophages. Very weak direct interactions between tumor cells and CD8+ T cells were noted. Similarly, in mIDH1 iCCA, the strongest interactions were between tumor cells and αSMA+ fibroblasts, as well as tumor cells and CD68+/CD163- macrophages (Figure 4B).

**FIGURE 5:**
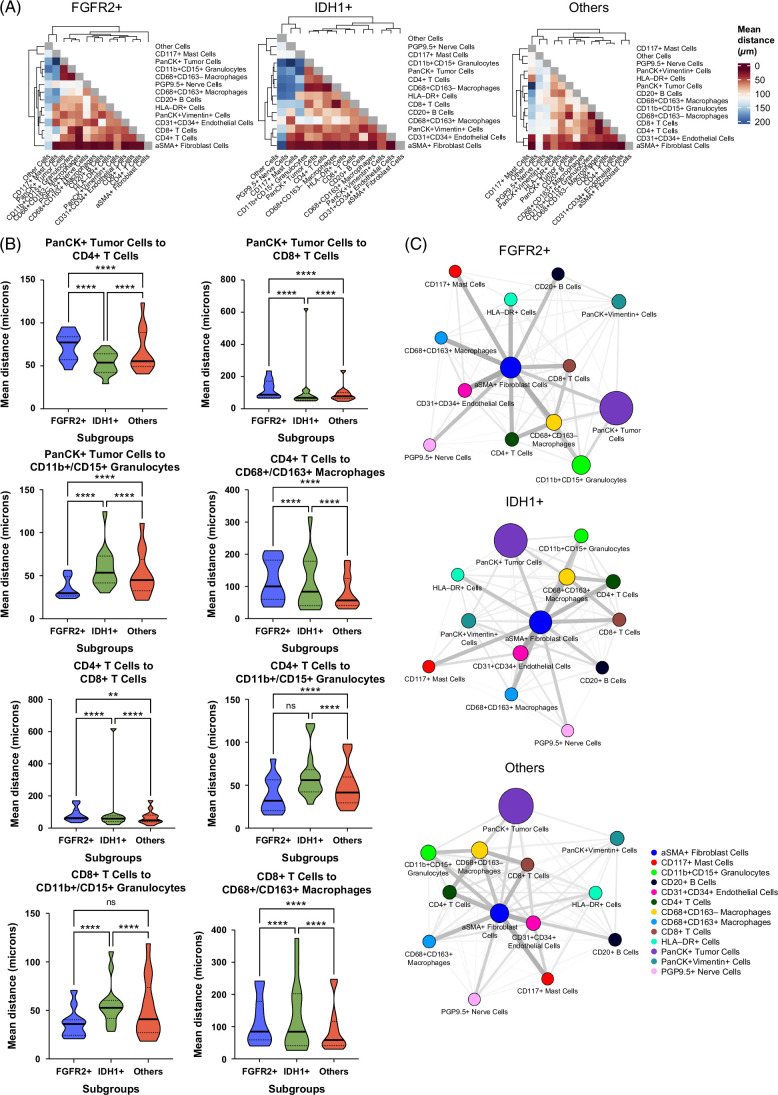
Comparisons of spatial distances between tumor cells and immune cell subtypes. (A) Heatmap showing the average spatial distance between cells of two specific subtypes. (B) Most important minimum Euclidean distances from each index cell type to other cell types are shown at the per-cell level as violin plots across molecular subtypes. P-values from Tukey’s multiple comparisons tests. (C) Network plots showing average cluster-to-cluster distance and cluster proportion, by tumor subtype. Each node represents a different cell type. Edge thickness and darkness is proportional to the mean distance between each cell type, and node size is proportional to the frequency of each cell type. Other cells have been excluded for the sake of visualization. Statistically significant *P* value is shown as follows: ** means *p*<0.01, **** indicates *p*<0.001. FGFR2 (blue), IDH1 (green), other (red). Abbreviations: aSMA, anti-smooth muscle antibody; FGFR, fibroblast growth factor receptor; HLA, human leukocyte antigen; IDH, isocitrate dehydrogenase; PanCK, pan-cytokeratin; PGP, protein gene product.

Finally, we quantified the distance between discrete cell subsets at the micron level by computing Euclidean distances from cells of a selected cell type to their nearest neighbors of another cell type and compared the mean distances (Figure 5). We first generated a heatmap of the mean distances between cell types in the 3 molecular-defined iCCA subgroups to qualitatively identify differences (Supplemental Figure S9, http://links.lww.com/HC9/B887). As expected, given their generally relatively high abundance, αSMA+ fibroblasts, CD68+/CD163- macrophages, and CD31+/CD34+ endothelial cells were near each other, tumor cells, and T cells across all molecular subgroups examined (Figure 5A). Here, we highlight a few specific spatial relationships that differed significantly across the molecular subgroups. Following our observation of increased CD11b+/CD15+ granulocytes in FGFR2+ iCCA, we specifically examined the spatial relationships of CD11b+/CD15+ granulocytes in FGFR2+ tumors. The distance between CD11b+/CD15+ granulocytes and tumor cells, as well as both CD8+ and CD4+ T-cell subsets, was reduced as compared to IDH1+ iCCA and FGFR2 WT/IDH1 WT iCCA (*p*<0.001 for all comparisons). FGFR2+ tumors also demonstrated separation between both CD4+ T cells and CD8+ T cells and tumor cells as compared to both IDH1+ iCCA and FGFR2 WT/IDH1 WT (*p*<0.001) (Figure 5B). Conversely, IDH1+ iCCA had closer spatial interactions between CD4+ T cells and tumor cells as compared to the other molecularly defined groups (*p*<0.001). CD4+ T cells have both tumor-promoting and tumor-rejecting functions, the latter largely through providing help to CD8+ T cells. Thus, we specifically examined the distance between CD4+ T cells and CD8 T cells in the IDH1+ TME and observed a higher distance than in any other molecular subset (*p*<0.001). IDH1+ tumors also demonstrated closer spatial interactions between CD68+/CD163+ macrophages and multiple cell subsets (CD20+ B cells, PanCK+/Vimentin+ cells, protein gene product 9.5+ nerve cells, and endothelial cells) than both FGFR2+ iCCA and FGFR2 WT/IDH1 WT (*p*<0.001) (Figure 5B and Supplemental Figures S10–S12, http://links.lww.com/HC9/B887).

To better understand how each cell type may be spatially related to one another, we generated a simplified network of cell types based on the minimum Euclidean distances from each cell type to all other cell types (Figure 5C). This technique emphasizes the shortest paths between cell types, suggesting closer functional or spatial relationships. In FGFR2+ TMEs, we confirmed that the node in the network associated with tumor cells was generally distant from the CD8+ T-cell effector immune cell types and near CD11b+/CD15+ granulocytes and CD68+/CD163+ macrophages. Notably, both CD8+ and CD4+ T cells were surrounded by macrophage nodes in IDH1 tumors and closer to each other. αSMA+ fibroblasts were close to tumor cells in both FGFR2+ and IDH1+ tumors, compared to other subtypes, highlighting their potential roles in mediating interactions among the included cell types.

## DISCUSSION

Although CCA is a relatively rare form of primary liver cancer accounting for 5%–10% of all primary liver malignancies, the overall incidence and mortality burden has been increasing over the past few decades. CCA is often diagnosed at an advanced stage, limiting therapeutic options, and resulting in a generally poor prognosis.^1^,^2^

The addition of an ICI targeting the PD-1 pathway in combination with platinum-based chemotherapy is the new standard of care for advanced CCA, but clinical outcomes remain poor. ICIs used in the TOPAZ and KEYNOTE-966 trials were durvalumab and pembrolizumab, respectively.^31^,^32^ Furthermore, pretreatment and post-treatment biopsies were not routinely collected in these studies and therefore little is known about the mechanisms of response and resistance to this frontline treatment combination. In the face of modest benefits and the lack of predictive biomarkers, it is essential to understand mechanisms of response and resistance to current combinations to identify better treatment strategies. CCAs are highly heterogeneous at both the intertumoural and intratumoural levels. The complex interaction between TME and the molecular alterations that contribute to this heterogeneity represents a limitation for common therapeutic strategies.

Our study aimed to deeply characterize the TME of iCCA and to identify unique features of iCCA with IDH1 or FGFR2 molecular alterations using automatic delineation of cell types from multidimensional marker expression and positional data generated by CODEX. We found that iCCAs are broadly characterized by an abundance of cancer-associated fibroblasts and immunosuppressive cell types including TAMs, and a relatively low abundance of T cells. This finding is consistent with prior reports from multiple groups and provides context for the low response rate of CCAs to ICI monotherapy.^33^,^34^,^35^

We also observe distinct features of the TME of iCCA with FGFR2 pathogenic fusions or rearrangements, including a lower abundance of CD8+ T cells as compared to FGFR2 WT iCCA. Our data are consistent with other studies that identified a relationship between FGFR2 molecular alterations and low immune infiltration at the transcriptional level.^22^,^36^,^37^ The mechanisms underlying the CD8+ T cell desert phenotype in FGFR2+ tumors have not been completely elucidated. It is proposed that FGFR alterations may inhibit T-cell infiltration and expansion via the local inhibition of interferon-γ and GZMB,^38^,^39^,^40^ although our results showed that CD4+ T cells in mFGFR2 tumors tended to have increased GZMB expression compared to other molecular subtypes. It has been hypothesized that although GZMB is typically associated with cytotoxic functions, in CD4+ T cells it may possess noncytotoxic functions such as regulating proinflammatory cells.^41^ As ICI responses are dependent upon the presence and modulation of various antitumor immune cells in the TME, including CD8+ T cells, our findings provide initial evidence that iCCA with FGFR2 alterations may be a particularly immune-resistant subtype of iCCA and also may inform the development of novel combination strategies with agents that facilitate the induction of T cells into tumors. One potential strategy is to use FGFR inhibitors to reprogram the TME via inhibition of FGFR signaling, which is supported by several preclinical studies.^37^,^42^ For instance, Wu and colleagues showed that the use of the FGFR2 inhibitor erdafitinib increased T-cell infiltration in immunocompetent mouse models of triple-negative breast cancer. Furthermore, the authors observed that FGFR inhibition combined with immune checkpoint blockade therapy greatly improved the therapeutic response of TNBC tumor models.^42^ Other studies in NSCLC have also shown that FGFR2 inhibition drives the expansion of T-cell clones while reducing other immunosuppressive cell subtypes to support enhanced antitumor immunity.^37^ None of the samples in our cohort had received prior FGFR2-directed therapy, and therefore, we are unable to evaluate whether treatment with an FGF2 inhibitor reprograms the TME. At the time of this report, FGFR2 inhibitors are used in the second-line treatment of iCCA, generally only after treatment with immunotherapy. Future research is needed to evaluate the combination of an FGFR2 inhibitor with an ICI in iCCA and determine if administering FGFR2 inhibitors prior to or concomitant to immunotherapy may act as an adjuvant to target T-cell activation and enhance clinical benefit.

We also showed a higher abundance of CD11b+/CD15+ granulocytes in FGFR2+ iCCA, which are subclassified as polymorphonuclear myeloid-derived suppressor cells (PMN-MDSC). We hypothesize that these PMN-MDSC may contribute to the low CD8+ T-cell infiltration observed in this subset of iCCA. Prior studies have found that PMN-MDSCs include local immune suppression that suppresses local T-cell antitumor activity and may drive T-cell exclusion.^37^ High-dimensional spatial maps and cellular interaction analysis also demonstrated closer proximity between PMN-MDSCs and tumor cells, CD4+ T cells, and CD8+ T cells in FGFR2 iCCA as compared to other subsets, providing additional evidence that PMN-MDSCs are a major contributor to induce local immune suppression in FGFR2 iCCA. Given the challenge of differentiating PMN-MDSC from neutrophils, further functional evaluation is needed to confirm their immunosuppressive function in iCCA^43^ and to determine if PMN-MDSCs could be subdivided into smaller populations with specific functional characteristics and/or if they represent a discrete single population distinct from classical neutrophils. Additional work is needed to understand the specific cross talk signals through which FGFR2 signaling in iCCA drives infiltration with PMN-MDSCs.

mIDH1 iCCAs also exhibited unique TME features. The most abundant cell types within the mIDH1 TME were TAMs and cancer-associated fibroblasts, which may form a physical barrier limiting therapeutic access as well as forming a complex signaling axis between neoplastic and stromal cells.^34^ Furthermore, we observed closer spatial association between CD8+ T cells and immunosuppressive M2-like CD68+/CD163+ macrophages in mIDH1 tumors compared to FGFR2+ iCCA. Prior work from our group has identified that the proximity of immunosuppressive TAMs and T cells may be an important determinant of response to immunotherapy in liver cancers.^44^ A poorly immunogenic TME characterized by the abundance of immunosuppressive innate immune cells such as TAMs and MDSCs affects ICI response, and CCA murine models showed that increased recruitment of PDL1+ TAMs was associated with decreased CD8+ T-cell infiltration. In addition, the blockade of TAMs and MDSCs potentiated the effect of ICI with anti-PD1 in preclinical models.^45^,^46^,^47^

In general, one major mechanism of CD8+ T-cell suppression by TAMs is the depletion of metabolites essential for T-cell proliferation, such as l-arginine, which is depleted by TAM-derived arginase-1 in various cancers.^48^ Although TAMs secreting arginase-1 are recognized to induce tumor immunosuppression in a wide variety of settings, it is not known whether TAM-T-cell interactions drive resistance to anti-PD1 plus chemotherapy in patients with CCA. Inhibiting the IDH pathway may be one way to reverse the immune inactivation and may lead to better responses to immune checkpoint therapy. Ivosidenib is an IDH1 inhibitor that showed some improvement in OS as monotherapy compared to placebo in the ClarIDHy trial.^14^ However, the response rates were limited. In preclinical models, pharmacologic mIDH1 inhibition stimulates CD8+ T-cell recruitment and IFNγ response genes, leading to a synergistic therapeutic effect of mIDH1 inhibitors and immune checkpoint blockade.^49^ Similar to our conclusion with FGFR2, combination therapy with an IDH1 inhibitor and ICI may improve response rates and should be studied further.

This work has several innovative conceptual and technical features. We were able to demonstrate the applicability of a polymerase-driven, highly multiplexed fluorescence-based imaging technology in the evaluation of primary liver cancers. While prior studies have sought to characterize the TME of iCCA, there has been limited analysis to stratify by molecular subtype.^33^ Other studies have focused on identifying niche features of the TME, but most studies have been done at the RNA level rather than at a protein level in human sample tissue.^22^,^50^ However, several limitations apply to this study. First, our sample size was limited and derived from only two centers in the United States, potentially limiting the generalizability of our findings as well as our ability to detect smaller signals between the molecular subsets. Second, due to the study’s retrospective nature, survival comparisons between the molecular subsets were exploratory in nature. Third, the analysis was limited by the lack of extensive functional markers to better subdivide cell populations based on their functional status, ie, the expression of markers that determine pro-immune “M1” (eg, CD40, CD80) and immunosuppressive “M2” (eg, arginase, CD206) functional states in monocytes/macrophages. Further work is warranted to dissect this complex and continuous cross talking, as an effective antitumor immune response against CCA will require identifying the best immune modulators that reprogram inhibiting signals to become signals that facilitate trafficking and function of the highest quality T cells in the TME.

In conclusion, our study demonstrates the feasibility of polymerase-driven highly multiplexed visualization of antibody binding events to distinguish single cells as well as tissue sections to achieve sophisticated immune profiling of iCCA tissue samples. Our study identifies differences in the TME between iCCAs defined by molecular characteristics, informing the eventual development of molecularly defined combinatorial immunotherapy.

## Supplementary Material
