# Supplementary material for: Deep immune profiling of intrahepatic cholangiocarcinoma with CODEX multiplexed imaging
Source: Hepatol Commun. 2025 Feb 19;9(3):e0632. doi: 10.1097/HC9.0000000000000632 (PMC11841852; doi:10.1097/HC9.0000000000000632)
Supplement: Supplementary file 1 [file hc9-9-e0632-s001.pdf]

Cholangiocarcinoma Supplementary Figures and Tables

Table 1. CODEX Standard 38-plex panel for Human FFPE			
CD107a<br>CD117<br>CD11b<br>CD134<br>CD14<br>CD141<br>CD15<br>CD163<br>CD183<br>CD197	CD20<br>CD21<br>CD31<br>CD34<br>CD3e<br>CD4<br>CD45<br>CD45RA<br>CD45R0<br>CD56	CD68<br>CD8<br>GATA3<br>HLADR<br>Granzyme B<br>ICOS<br>IDH1<br>PD-L1<br>PD-1<br>AXL	PGP9.5,<br>PanCK<br>Podoplanin<br>SPP1<br>Siglec8<br>Tbet<br>Vimentin<br>aSMA

Supplementary Table 1. CODEX Standard 38-plex panel for Human FFPE.
Abbreviations: FFPE, formalin-fixed paraffin-embedded

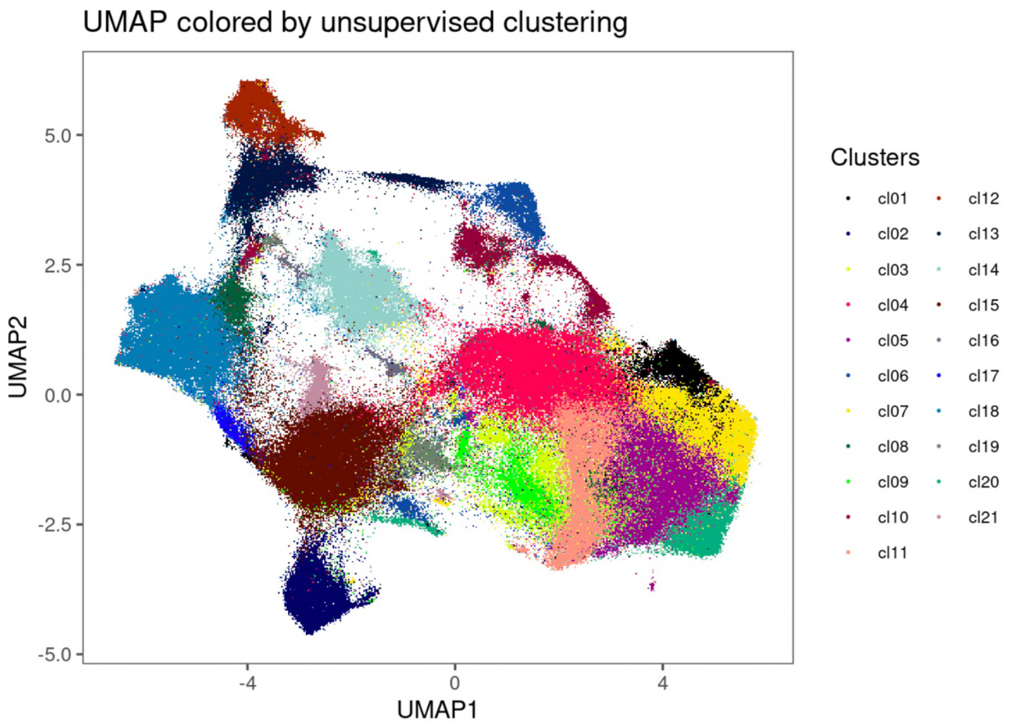

Supplementary Figure 1. UMAP diagram colored by unsupervised clustering.

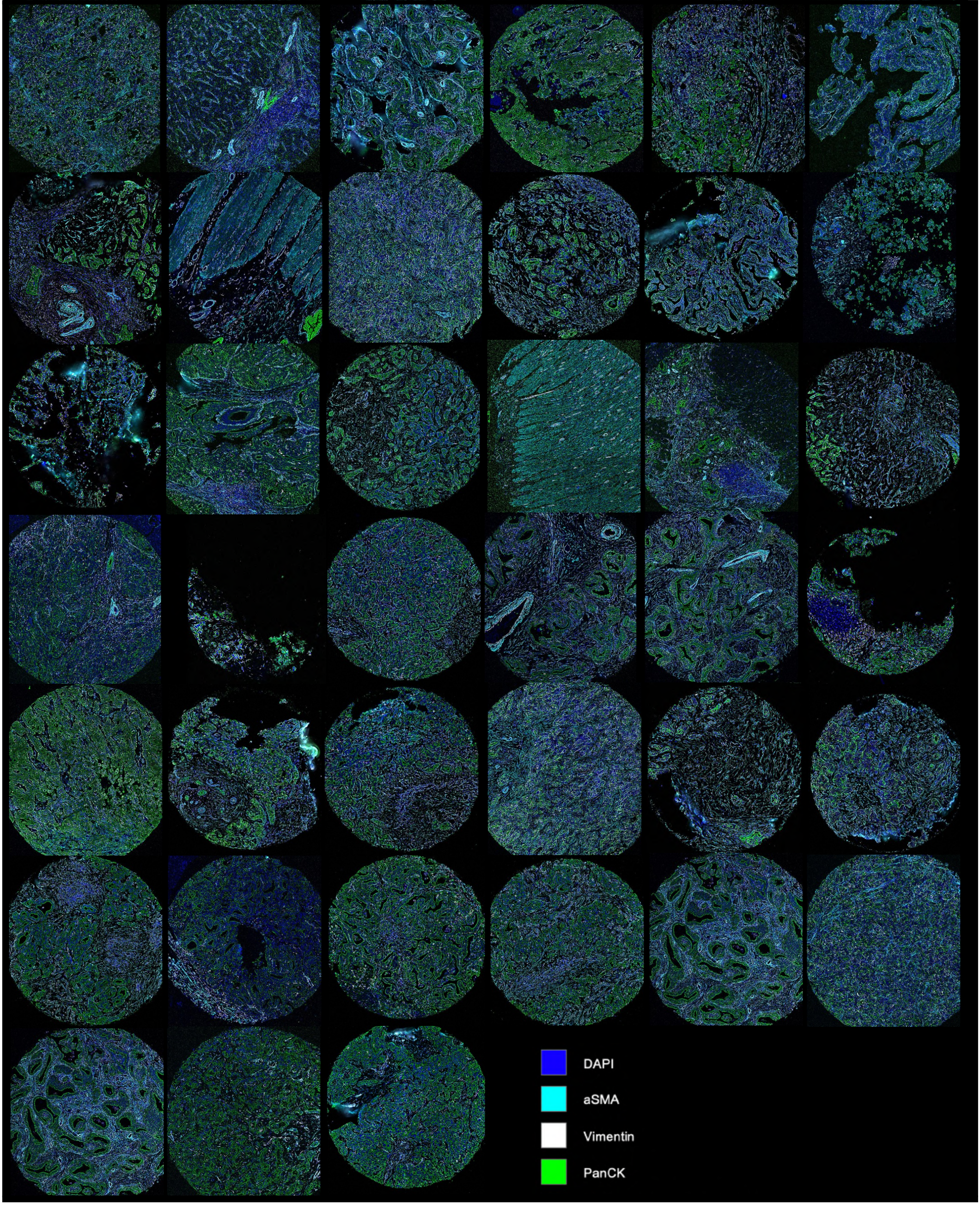

Supplementary Figure 2. A representative CODEX image from each of the 38 samples collected from our 24 patients. Most patients had one sample collected and a few patients had two samples collected. One patient had three samples collected.

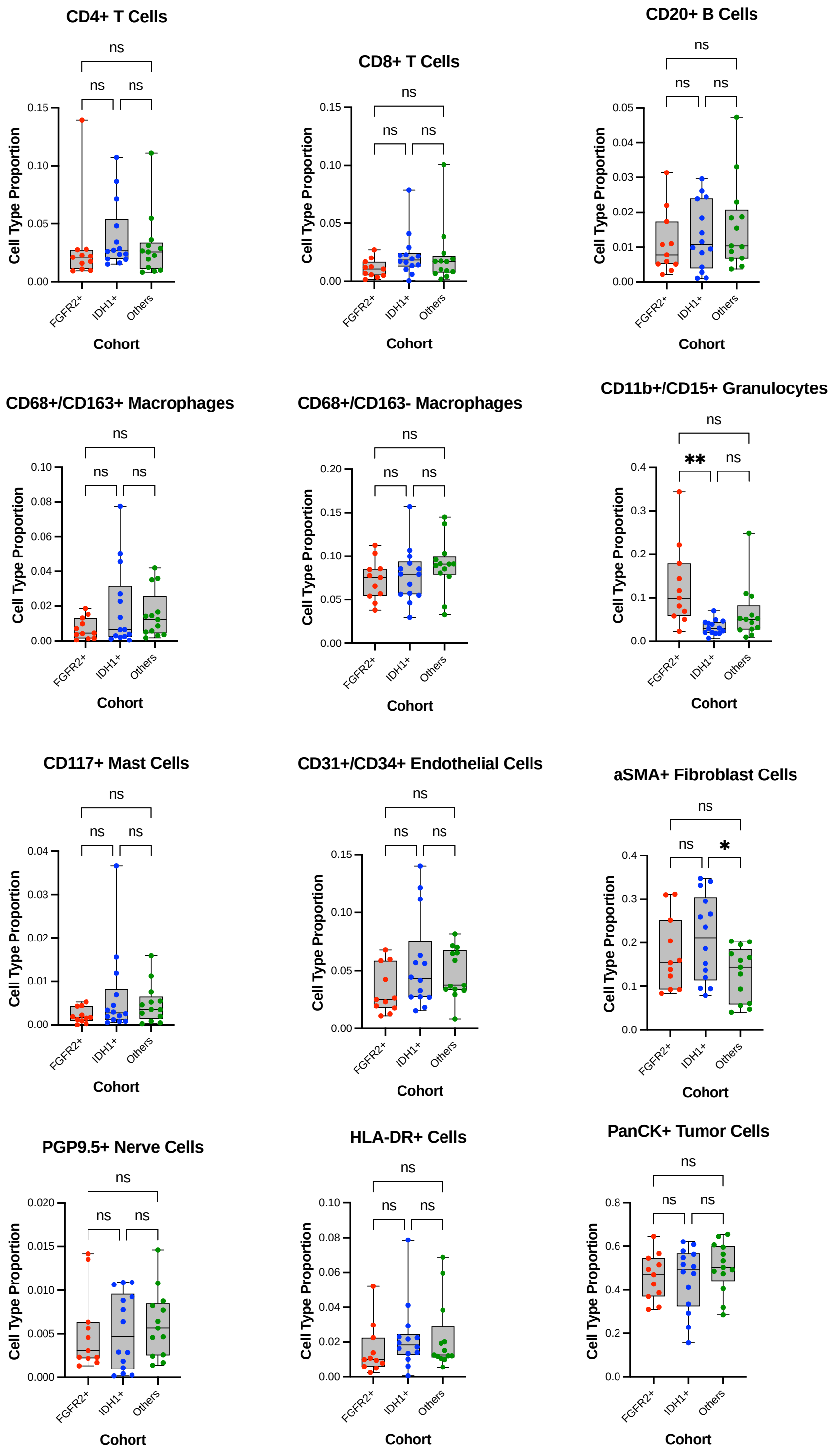

Supplementary Figure 3. Quantification of immune cells in the TME of molecular subtypes. Box and whisker plots comparing the proportion of cell subtypes in the tumor subtypes. P-values from one-way ANOVA test. * indicates $p < 0.05$

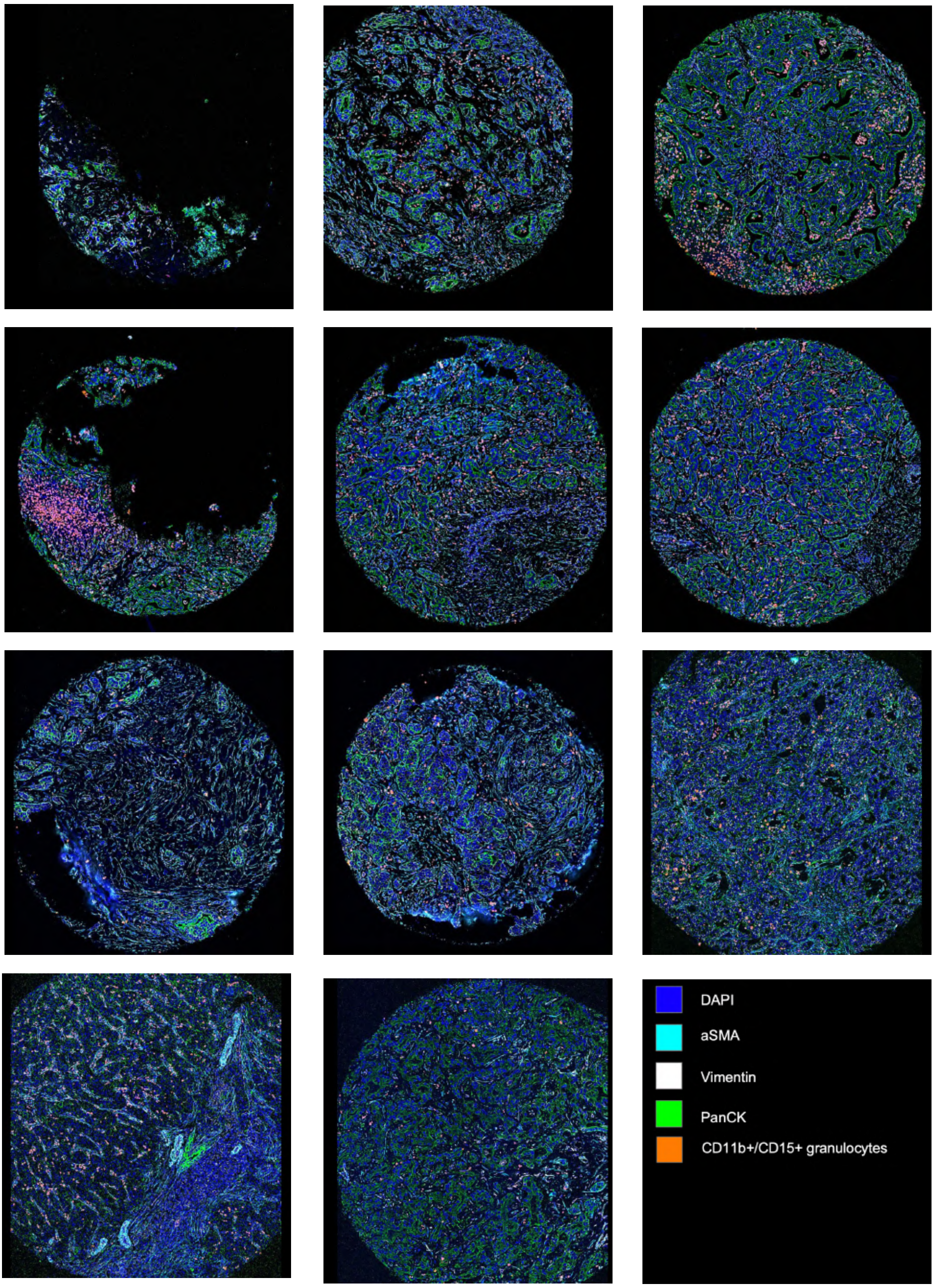

Supplementary Figure 4. A representative CODEX image for each of the FGFR2+ samples highlighting high CD11b+/CD15+ granulocytes.

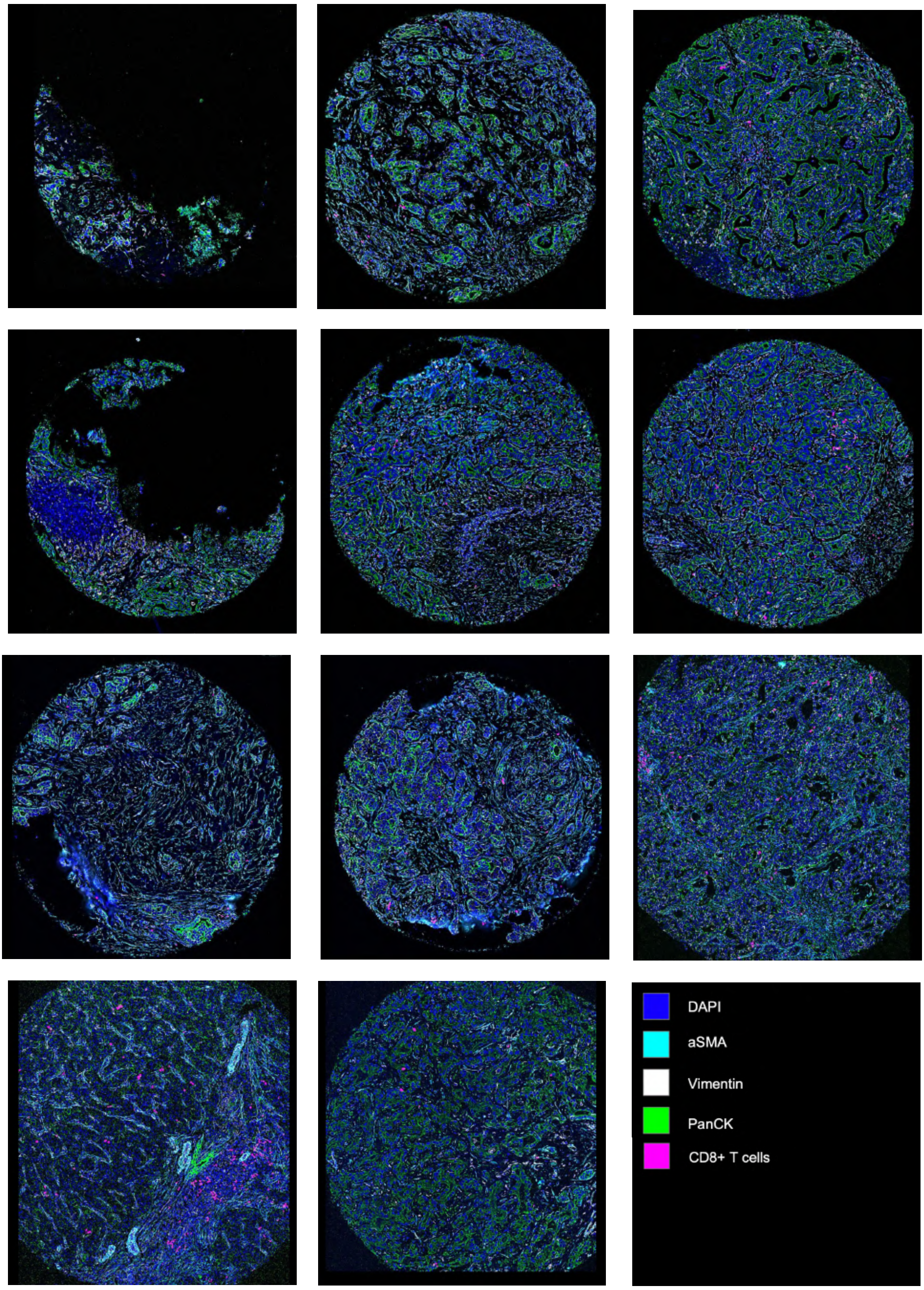

Supplementary Figure 5. A representative CODEX image for each of the FGFR2+ samples highlighting low CD8+ T cells.

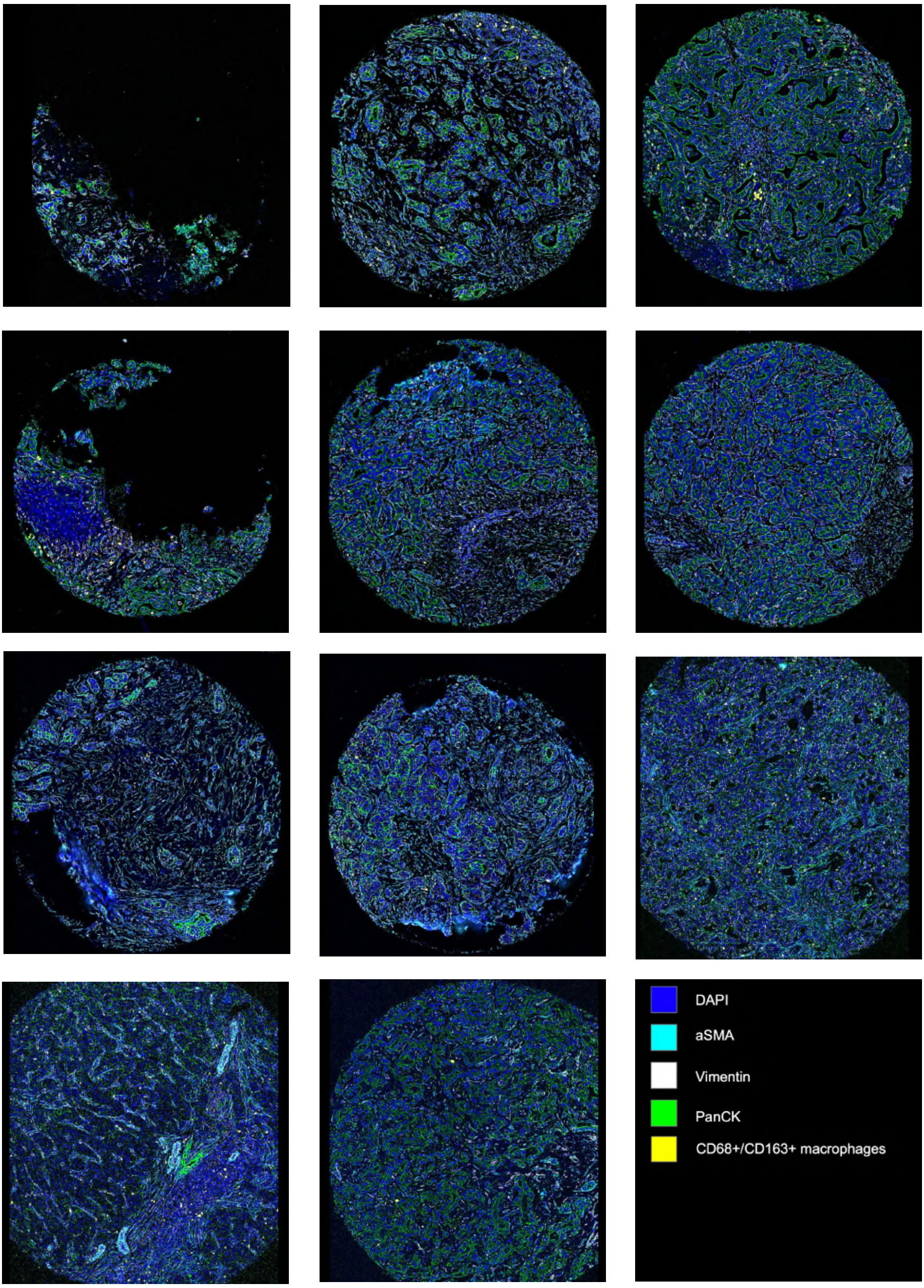

Supplementary Figure 6. A representative CODEX image for each of the FGFR2+ samples highlighting low CD68+/CD163+ macrophages.

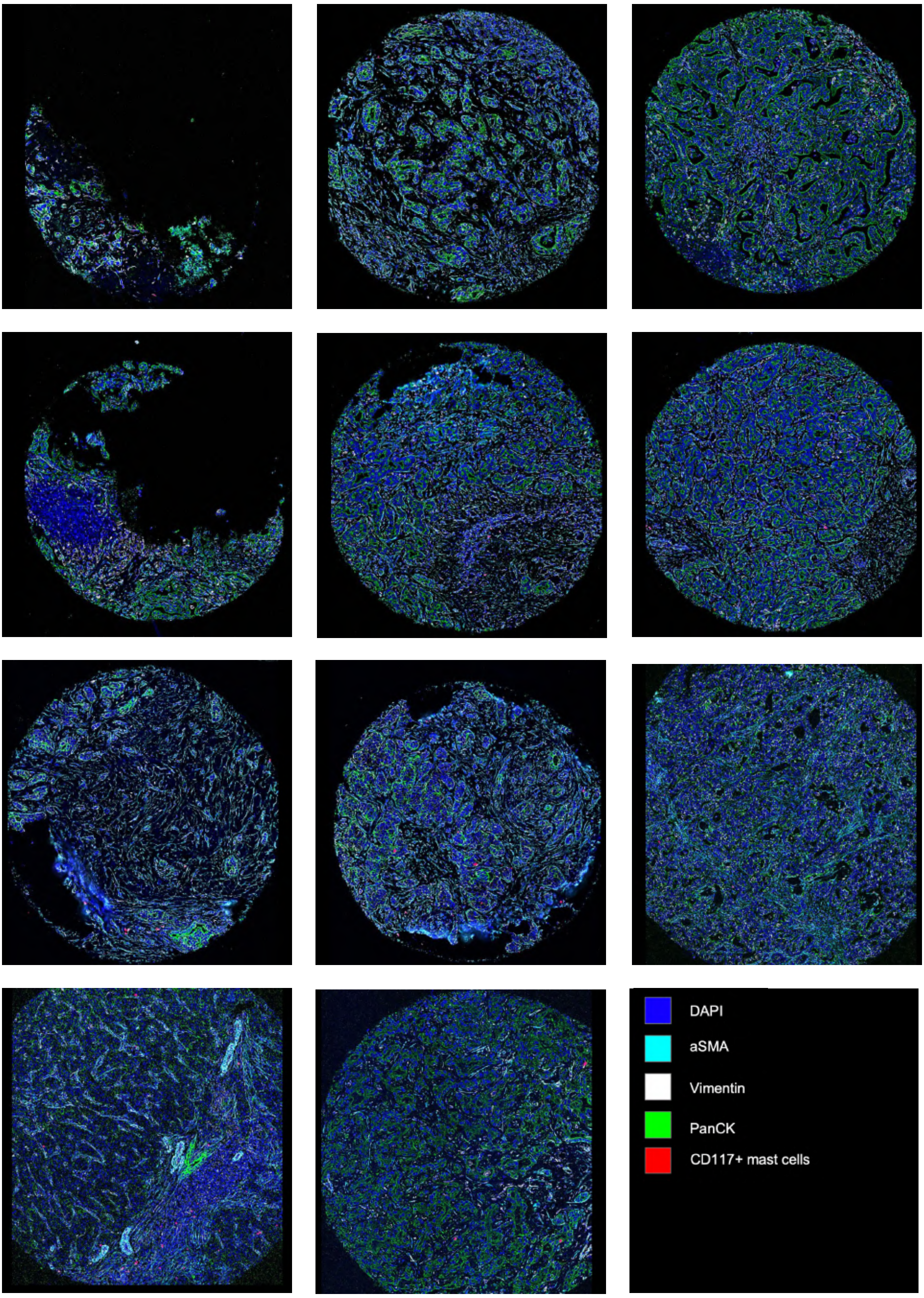

Supplementary Figure 7. A representative CODEX image for each of the FGFR2+ samples highlighting low CD117+ mast cells.

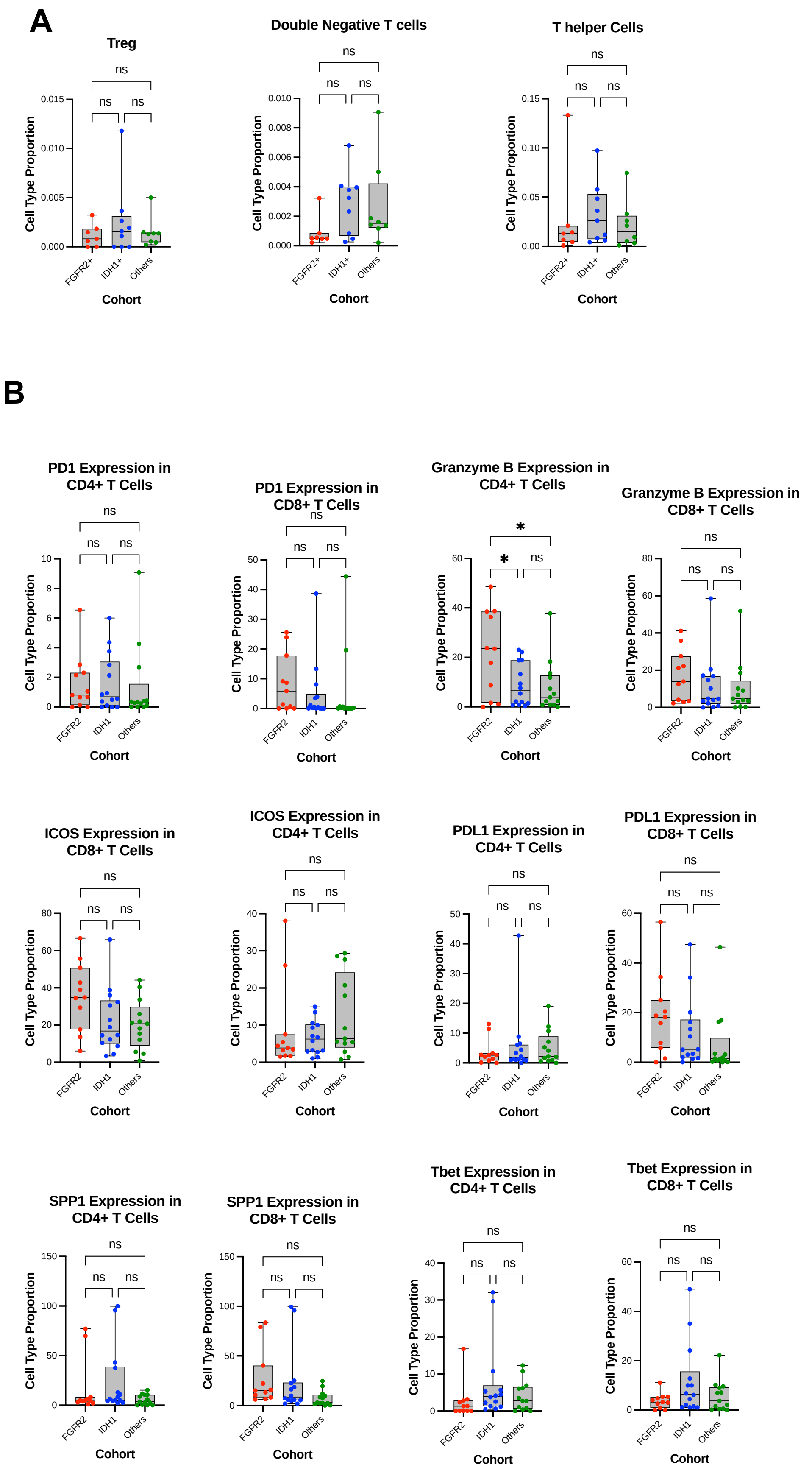

Supplementary Figure 8. A) Boxplots examining the frequency of T cell subsets within the three subgroups. B) Boxplots examining the frequency of biomarker expression in CD4+ and CD8+ T cells across the three subgroups. * means $p < 0.05$

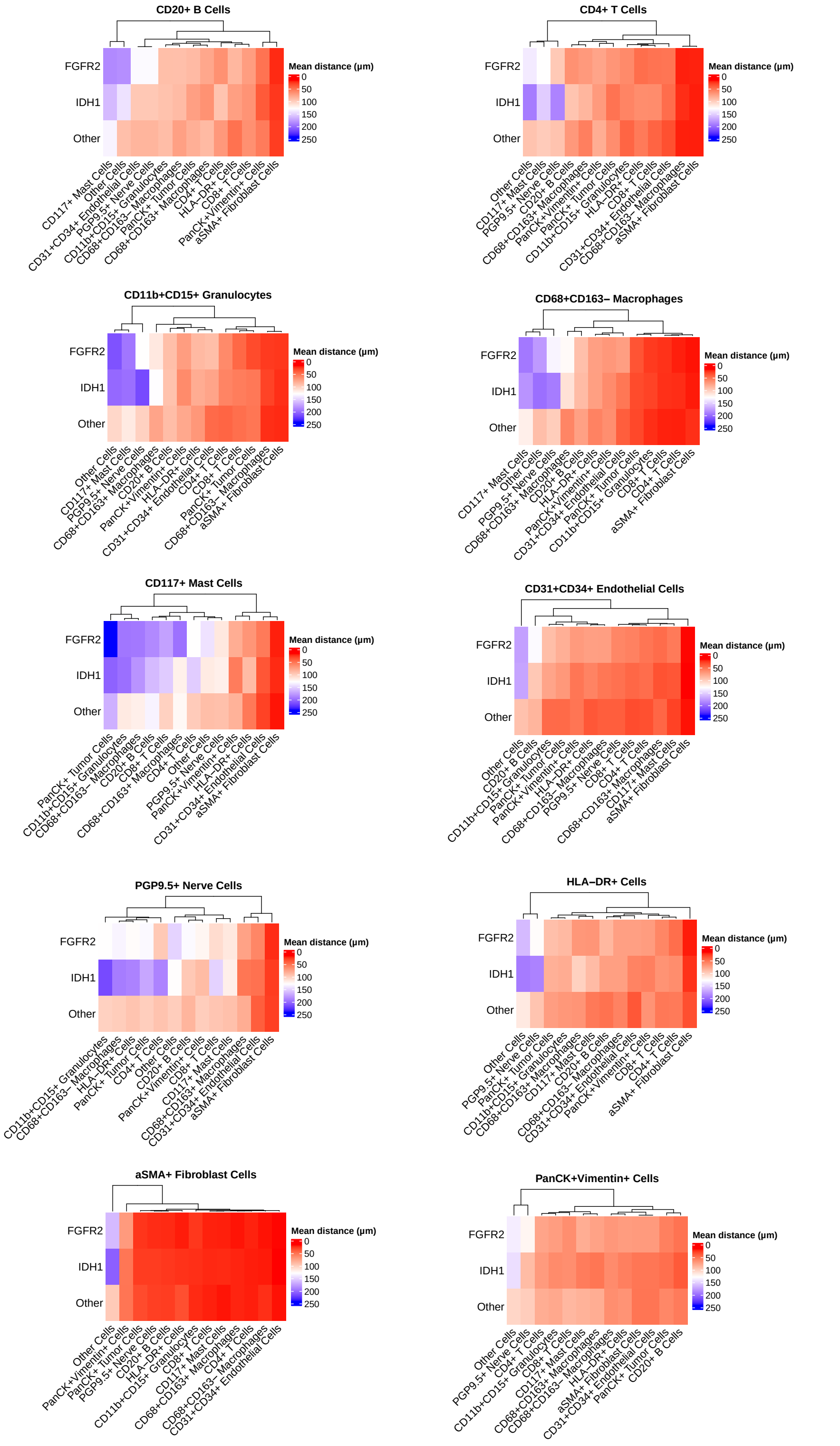

Supplementary Figure 9. The heatmap of spatial analysis among the cell types the three subgroups – FGFR2+, IDH1+ and others.

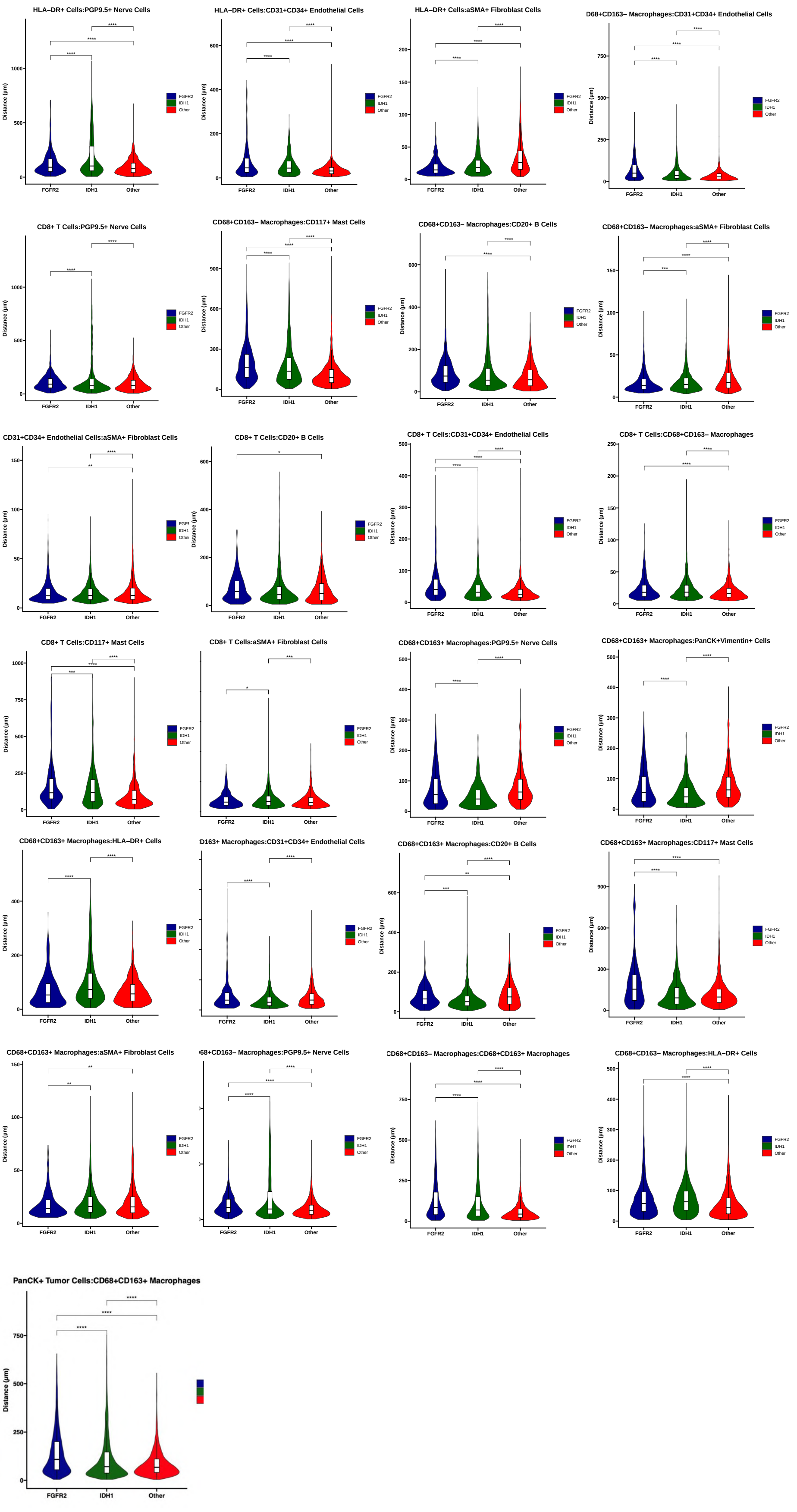

Supplementary Figure 10. Violin plots comparing the mean distance between cellular subsets across molecular subtypes. P-values from Tukey's multiple comparisons tests. Statistically significant P value is shown as follows: * means $p < 0.05$, ** means $p < 0.01$, *** means $p < 0.001$, **** indicates $p < 0.0001$. FGFR2 (blue), IDH1 (green), other (red).

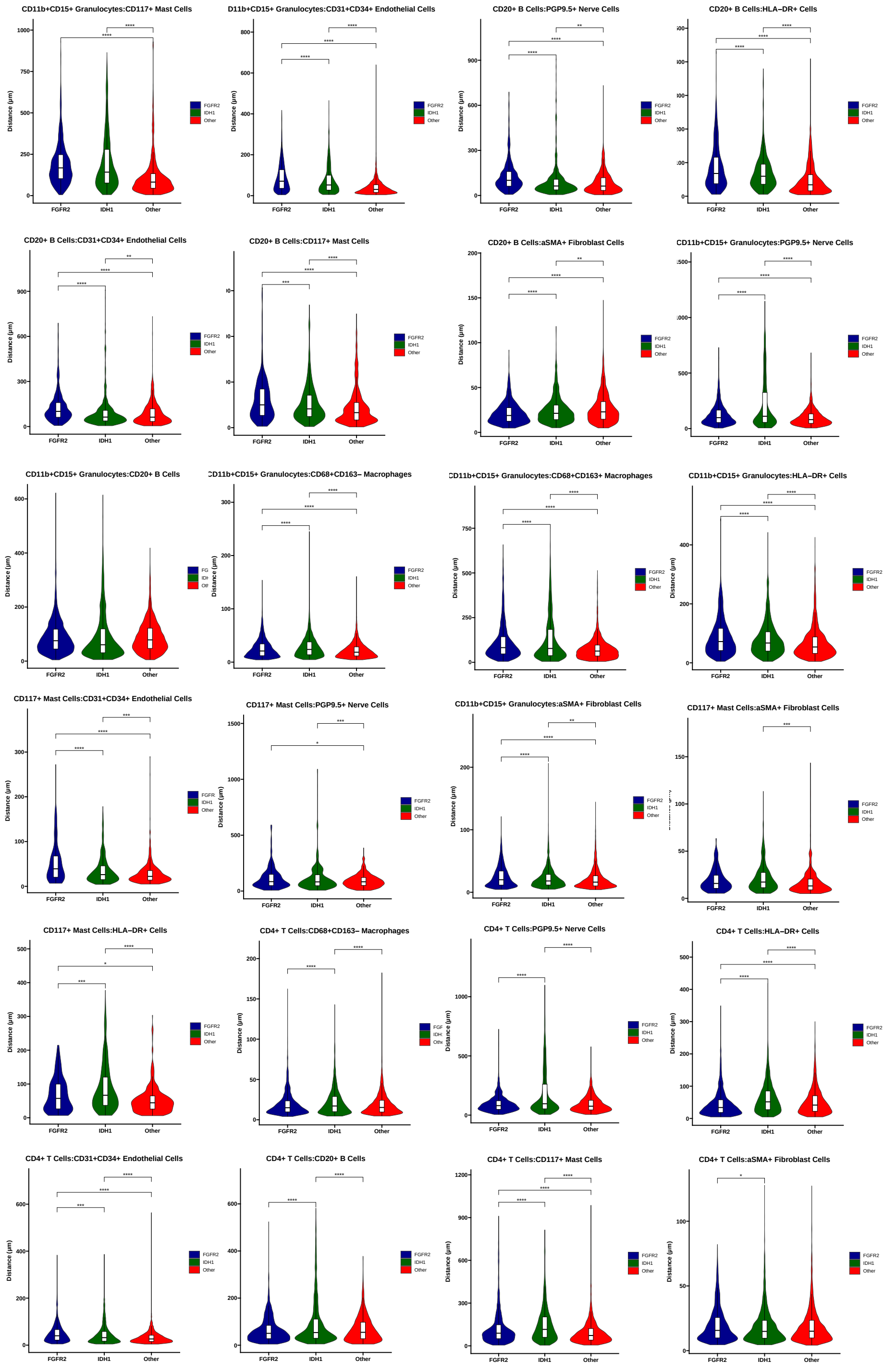

Supplementary Figure 11. Violin plots compare the mean distance between cellular subsets across molecular subtypes. P-values from Tukey's multiple comparisons tests. Statistically significant P value is shown as follows: * means $p < 0.05$, ** means $p < 0.01$, *** means $p < 0.001$, **** indicates $p < 0.0001$. FGFR2 (blue), IDH1 (green), other (red).

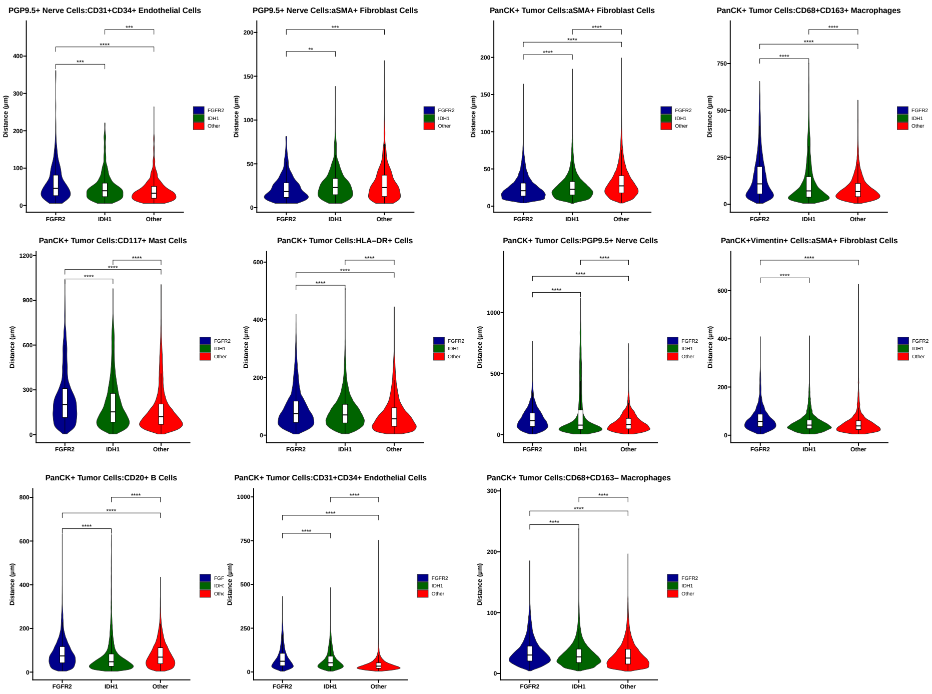

Supplementary Figure 12. Violin plots comparing the mean distance between cellular subsets across molecular subtypes. P-values from Tukey's multiple comparisons tests. Statistically significant P value is shown as follows: * means p < 0.05, ** means p < 0.01, *** means p < 0.001, **** indicates p < 0.0001. FGFR2 (blue), IDH1 (green), other (red).
